# Fursultiamine Prevents Drug-Induced Ototoxicity by Reducing Accumulation of Reactive Oxygen Species in Mouse Cochlea

**DOI:** 10.3390/antiox10101526

**Published:** 2021-09-26

**Authors:** Ye-Ri Kim, Tae-Jun Kwon, Un-Kyung Kim, In-Kyu Lee, Kyu-Yup Lee, Jeong-In Baek

**Affiliations:** 1 Department of Biology, College of Natural Sciences, Kyungpook National University, Daegu 41566, Korea; yell_90@knu.ac.kr (Y.-R.K.); kimuk@knu.ac.kr (U.-K.K.); 2 Advanced Bio-Resource Research Center, Kyungpook National University, Daegu 41566, Korea; 3 Laboratory Animal Center, Daegu-Gyeongbuk Medical Innovation Foundation (DGMIF), Daegu 41566, Korea; tjkwon@dgmif.re.kr; 4 School of Life Sciences, KNU Creative BioResearch Group (BK21 Plus Project), Kyungpook National University, Daegu 41566, Korea; 5 Department of Internal Medicine, Research Institute of Aging and Metabolism, School of Medicine, Kyungpook National University, Daegu 41566, Korea; leei@knu.ac.kr; 6 Department of Otorhinolaryngology-Head and Neck Surgery, School of Medicine, Kyungpook National University, Daegu 41566, Korea; 7 Department of Aroma-Applied Industry, College of Herbal Bio-Industry, Daegu Haany University, Gyeongsan 38610, Korea

**Keywords:** cisplatin, kanamycin, ototoxicity, reactive oxygen species, fursultiamine

## Abstract

Drug-induced hearing loss is a major type of acquired sensorineural hearing loss. Cisplatin and aminoglycoside antibiotics have been known to cause ototoxicity, and excessive accumulation of intracellular reactive oxygen species (ROS) are suggested as the common major pathology of cisplatin- and aminoglycoside antibiotics-induced ototoxicity. Fursultiamine, also called thiamine tetrahydrofurfuryl disulfide, is a thiamine disulfide derivative that may have antioxidant effects. To evaluate whether fursultiamine can prevent cisplatin- and kanamycin-induced ototoxicity, we investigated their preventive potential using mouse cochlear explant culture system. Immunofluorescence staining of mouse cochlear hair cells showed that fursultiamine pretreatment reduced cisplatin- and kanamycin-induced damage to both inner and outer hair cells. Fursultiamine attenuated mitochondrial ROS accumulation as evidenced by MitoSOX Red staining and restored mitochondrial membrane potential in a JC-1 assay. In addition, fursultiamine pretreatment reduced active caspase-3 and TUNEL signals after cisplatin or kanamycin treatment, indicating that fursultiamine decreased apoptotic hair cell death. This study is the first to show a protective effect of fursultiamine against cisplatin- and aminoglycoside antibiotics-induced ototoxicity. Our results suggest that fursultiamine could act as an antioxidant and anti-apoptotic agent against mitochondrial oxidative stress.in cochlear hair cells.

## 1. Introduction

Acquired hearing loss is known as one of the most common sensory disorders. Approximately 6% of the world’s population currently has hearing loss, with more than 2 billion people predicted to have hearing loss by 2050 [1]. The major causes of acquired hearing loss include infection, trauma, noise, injury, and ototoxic drugs. Particularly, two major classes of ototoxic drugs induce permanent hearing loss: aminoglycoside antibiotics and platinum-based chemotherapeutic agents. Both types of drugs damage inner ear hair cells through accumulation of ROS that triggers a pro-inflammatory response cascade including caspase 3 activation [2], thus limiting their clinical application.

Cisplatin (CP), also known as *cis*-diamminedichloridoplatinum (II) or *cis*-[PtCl_2_NH_3_]_2_)], is one of the platinum-based chemotherapeutic agents. It is an effective anticancer treatment for solid and hematological malignancies such as lung, esophageal, bladder, breast, cervical, ovarian, and testicular cancers [3]. Upon its entry into a cell, CP creates a *cis*-geometry by forming inter- or intra-strand crosslinks with DNA bases. This binding damages nuclear and mitochondrial DNA, leading to inhibition of gene transcription and DNA replication and eventually resulting in apoptosis of cancer cells [4]. Nevertheless, CP also exerts nonspecific toxicity on normal cells, resulting in side effects such as neurotoxicity, hepatotoxicity, nephrotoxicity, and ototoxicity. Hearing loss occurs after CP administration in 11–97% cases, with an average incidence of 62% [5,6]. Numerous previous studies have suggested that CP triggers cytotoxicity in hair cells by inducing diverse intracellular responses, including direct DNA damage, endoplasmic reticulum stress-induced inflammation, autophagy, and especially excessive accumulation of reactive oxygen species (ROS) [7].

Aminoglycosides are a class of antibiotics that target prokaryotic cells. They inhibit protein synthesis through direct interaction to ribosomal RNA, which increase mRNA misreading. Consequently it causes accumulation of misfolded proteins, leading to various cellular stress [8]. Although eukaryotic and prokaryotic ribosomes have structural differences, mitochondrial ribosomes are known to be more related to each other, thus mammalian cells can also be targets of aminoglycoside antibiotics. Once aminoglycosides enter the cell, they accumulate within mitochondria and endoplasmic reticulum (ER), resulting in ER stress and mitochondrial damage [9]. The significant cytotoxicity of aminoglycosides has been reported in kidney (nephrotoxicity) and inner ear (cochleotoxicity and vestibulotoxicity).

Although CP and aminoglycosides are fundamentally different molecules and have distinct physicochemical properties, cochleotoxicity, which particularly damages hair cells, is a common side-effect of these agents. CP- and aminoglycoside-induced hair cell damages eventually result in progressive, bilateral, and irreversible sensorineural hearing loss [9]. Although the precise underlying mechanisms are still incompletely understood, it has been demonstrated that mitochondrial damage and excessive accumulation of ROS followed by collapse of the intracellular redox balance play a key role in both CP- and aminoglycoside ototoxicity [8,10,11,12]. It provides a strong possibility that prevention or rescue of oxidative stress could be an effective strategy to protect hair cells from drug-induced cellular damages.

Fursultiamine (FT), or thiamine tetrahydrofurfuryl disulfide, is a lipophilic thiamine derivative that increases thiamine bioavailability. Thiamine is a cofactor for multiple enzymes that are essential for cellular energy metabolism, including glycolysis and oxidative decarboxylase multi-enzyme complexes such as pyruvate dehydrogenase and α-ketoglutarate dehydrogenase. Especially it is essentially required as a coenzyme for branched-chain ketoacid and branched amino acid dehydrogenase complexes [13,14,15]. Transketolase, a reversible cytosolic enzyme that catalyzes the first and last step of the pentose phosphate pathway, is dependent on the cofactor thiamine diphosphate that plays a major role in the production of NADPH to maintain glutathione levels in the intracellular antioxidative system [16,17,18]. Consistently, several studies suggest that thiamine exerts antioxidant effects in mammals. [19,20].

Based on these previous findings, we hypothesized that FT treatment could prevent CP- or aminoglycoside-induced cochlear oxidative stress leading to ototoxicity. To test this hypothesis, we examined the protective effects of FT treatment on cochlear hair cell viability, apoptotic signaling, ROS levels, and mitochondria membrane potential in mouse cochlear explants treated with CP or aminoglycoside kanamycin (KM).

## 2. Materials and Methods

### 2.1. Organotypic Cochlear Explants

Postnatal day 3 Institute for Cancer Research (ICR) mice were purchased from Hyochang Science (Daegu, Korea) for cochlear explants. The dissected cochleae were incubated in a humidified atmosphere of 5% CO_2_ at 37 °C in culture medium composed of high-glucose Dulbecco’s Modified Eagle’s Medium (Hyclone, Logan, UT, USA) containing 10% fetal bovine serum (Hyclone) and ampicillin (10 μg/mL; Life Technologies, Carlsbad, CA, USA). Cultured specimens were divided into six groups: untreated control (CT), cisplatin alone (CP), CP with FT pretreatment (CP + FT), kanamycin alone (KM), KM with FT pretreatment (KM + FT) and FT alone (FT). In all experiments, two cochleae obtained from a mouse were assigned into different groups to minimize experimental errors due to individual differences. Three cultured explants were used for each group. After 16 h of incubation, specimens in the CP + FT, KM + FT, and FT groups were pretreated with 10 μM or 100 μM of FT in dimethyl sulfoxide (DMSO) diluted in culture medium. After 1 h of incubation, 30 μM CP (Ildong Pharmaceutical Co., Daegu, Republic of Korea) or 1.2 mM kanamycin sulfate (Thermo Fisher Scientific, Waltham, MA, USA) was added to the culture medium. The concentration of pretreated FT was optimized for each ototoxic drug.

A total of 120 male and female mice were used for our study, and all animal experiments were approved by the Use Committee on the Ethics of Animal Experiments and were performed following guidelines of the Institutional Animal Care and Use Committee of Kyungpook National University.

### 2.2. Phalloidin Staining

To assess the morphology of inner hair cells (IHCs) and outer hair cells (OHCs) in the organ of Corti, specimens were rinsed with phosphate-buffered saline (PBS) after 48 h of incubation, and were fixed with 4% paraformaldehyde (pH 7.4) in PBS for 15 min. After washing with PBS three times, it was stained with Alexa Fluor^®^ 488- or 555-conjugated phalloidin (1:1000; Invitrogen-Molecular Probes, Eugene, OR, USA) in PBS for 1 h at room temperature (RT). Finally, the specimens were mounted on glass slides using Fluoromount (Sigma-Aldrich, St. Louis, MO, USA) and visualized using an Axio Imager A2 fluorescence microscope (Carl Zeiss, Oberkochen, Germany).

### 2.3. Immunohistochemistry and Terminal Deoxynucleotidyl Transferase dUTP Nick End Labeling (TUNEL) Assay

To determine whether cell death in the organ of Corti occurred via apoptosis, we performed immunohistochemistry for active caspase-3 and TUNEL assay. After fixation, specimens were permeabilized with 0.1% Triton X-100 in PBS (PBS-Tx) for 30 min at RT and blocked with 5% normal goat serum for 1 h at RT. The specimens were incubated with anti-active caspase-3 (1:1000; Cell Signaling Technology, Beverly, MA, USA) diluted in blocking solution overnight. After three rinses with PBS, samples were labeled with Alexa Fluor^®^ 488-conjugated goat anti-rabbit immunoglobulin G (1:1000; Invitrogen, La Jolla, CA, USA) diluted in blocking solution for 1 h at RT. F-actin was labeled with Alexa Fluor^®^ 555-conjugated phalloidin in PBS-Tx for 1 h at RT.

To detect DNA fragmentation, a marker of apoptotic cell death, we used the TUNEL assay kit (Promega, Madison, WI, USA) following the manufacturer’s protocol. Specimens were fixed with 4% paraformaldehyde in PBS for 15 min at RT and rinsed with PBS. And then, they were permeabilized with 0.1% PBS-Tx in 0.1% sodium citrate for 30 min at 37 °C and stained with TUNEL working solution for 30 min at 37 °C. F-actin was labeled with Alexa Fluor 555-conjugated phalloidin in PBS-Tx for 3 h at RT in the dark. Specimens were mounted on glass slides using Fluoromount and visualized using a Zeiss Axio Imager A2 fluorescence microscope.

### 2.4. Examination of Mitochondrial ROS Levels

To measure mitochondrial ROS levels, cochlear explant samples were rinsed with PBS after 24 h of incubation and stained with 5 μM MitoSOX Red (Invitrogen-Molecular Probes) diluted in PBS for 5 min in a humidified atmosphere of 5% CO_2_ at 37 °C. Specimens were then washed with PBS and visualized using a Zeiss Axio Imager A2 fluorescence microscope.

### 2.5. Analysis of Mitochondrial Membrane Potential (ΔYm)

We used cationic fluorescent dye MitoProbeTm JC-1 (Invitrogen-Molecular Probes) to measure mitochondrial membrane potential. After 30 h of incubation, specimens were washed with PBS and stained with JC-1 (2 μM) for 2 h in 5% CO_2_ at 37 °C in the dark. Before JC-1 staining, 50 μM carbonyl cyanide 3-chlorophenyhydrazone (CCCP) was added to specimens not treated with any drugs to verify the sensitivity of JC-1 assay, and then the specimens were incubated for 5 min in 5% CO_2_ at 37 °C to activate CCCP. We split JC-1 fluorescence into red and green colors, and the split images were converted to grayscale and assessed using ImageJ software (http://imageJ.nih.gove/ij/, accessed on 4 January 2021).

### 2.6. Quantification of Hair Cell Survival and Statistical Analysis

For quantitative analysis of hair cell damages, IHCs and OHCs that have intact v-shaped stereocilia were individually counted among a basilar membrane length of 200 μm in the apex (30%), mid (50%), and base (70%) regions of each cochlear explant. Data were analyzed using two-tailed Student’s *t*-tests with *p*-values < 0.05 considered statistically significant.

## 3. Results

### 3.1. Fursultiamine Protects Hair Cells from Drug-Induced Damages

**Figure 1:**
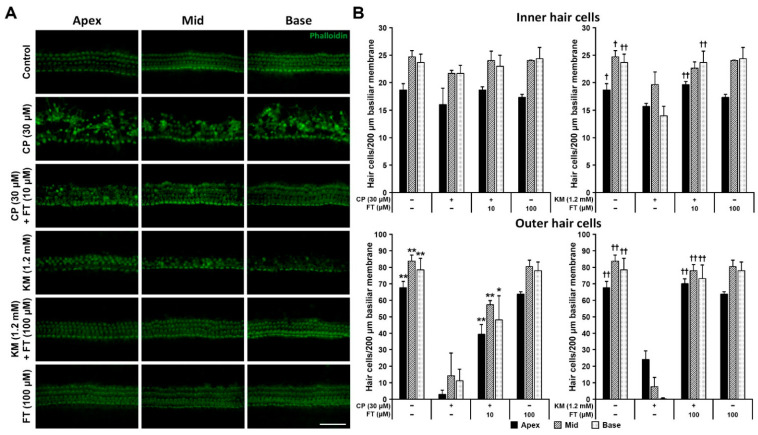
Effect of FT pretreatment on CP-, KM-induced ototoxicity in hair cells. (**A**) Images of fluorescence in mouse cochlear explants from Control, CP, CP + FT, KM, KM + FT, and FT groups (*n* = 3, each). Stereocilia bundles of IHCs and OHCs were stained with phalloidin (green). (**B**) Number of surviving IHCs (upper) and OHCs (lower) along a basilar membrane length of 200 μm in each region. All groups contained 9 mice. Data are shown as mean ± SD. *, ^†^ *p* < 0.05; **, ^††^ *p* < 0.01 (student’s *t*-tests) compared with the CP group. CP: cisplatin, KM: kanamycin, FT: fursultiamine. Scale bar, 50 μm.

To determine whether FT can protect hair cells from CP- or KM-induced ototoxicity, hair cell damages were compared between FT-pretreated and FT-untreated cochlear explants, under CP or KM administration. In the untreated CT group, IHCs (one row) and OHCs (three rows) showed intact arrangement from the apex to the base. However, both 30 μM CP and 1.2 mM KM disturbed the arrangement of both IHCs and OHCs (Figure 1A), with a more pronounced breakdown of stereocilia bundles in OHCs than in IHCs (Figure S1). To examine the protective effect of FT, we pretreated cochlear explants with 10 or 100 μM FT before CP or KM treatment each. Compared with the CP and KM only groups, two FT pretreated groups (CP + FT and KM + FT) displayed significantly less hair cell degeneration, suggesting protective effect of FT on drug-induced cytotoxicity of hair cells.

When this result was quantitatively analyzed by counting the number of surviving hair cells having v-shaped stereocilia, the protective effect of FT against CP- or KM-induced hair cell damages was statistically verified in all three cochlear regions, apical, middle and basal turns, especially in OHCs (Figure 1B). Also, 10 and 100 μM FT had no detrimental effects on IHCs and OHCs indicating that it did not induce cytotoxic responses in hair cells. Although there was no statistical significance in IHCs, especially in the CP-pretreated group, it seems to be due to resistance of IHCs against CP rather than that FT is not effective. Moreover, the histological damages that cannot be quantified, disarrangement of hair cell rows, cell-cell spacing, and/or hair cell disorientations, were remarkably restored by pretreatment with FT. Therefore, our result suggests that FT effectively prevents CP and KM-induced hair cell loss in cochlear tissue.

### 3.2. Fursultiamine Suppresses Activation of Apoptotic Hair Cell Death in Mouse Cochlear Explants

**Figure 2:**
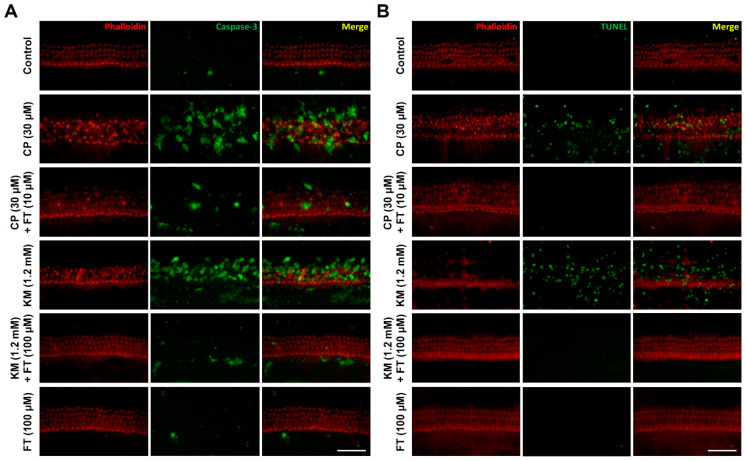
Effect of FT on CP- and KM-induced apoptosis of hair cells. Apoptosis and DNA damage were detected by (**A**) anti-active caspase-3 and (**B**) TUNEL assays, respectively, in Control, CP, CP + FT, KM, KM + FT, and FT groups (*n* = 9, each). Images were acquired from the mid region of the organ of Corti. Phalloidin (red) and active caspase-3 or TUNEL (green). A total of 30 mice were used in each experiment. CP: cisplatin, KM: kanamycin, FT: fursultiamine. Scale bar, 50 μm.

Since we have identified that FT prevents hair cells from CP and KM cytotoxic damage, we next investigated whether the protective effect of FT is due to suppression of apoptotic hair cell death. Immunohistochemical staining of cleaved caspase-3, final executioner of intrinsic apoptosis, and detection of DNA fragmentation were performed to detect activation of the apoptosis signaling pathway. As a result, while the organ of Corti. treated with CP or KM showed markedly increased levels of cleaved caspase-3 compared to untreated control, FT pretreatment before CP or KM exposure decreased the caspase-3 signal (Figure 2A). Consistently, the number of TUNEL-positive cells in which apoptotic DNA fragmentation occurred was also markedly decreased almost to the control level by FT pretreatment than those of only CP- or KM-treated organ of Corti (Figure 2B). Thus, these results suggest that CP- or KM-induced hair cell damages resulted in apoptotic cell death, and it was effectively inhibited by FT administration in mouse cochlea.

### 3.3. Fursultiamine Decreases Oxidative Stress and Prevents Disruption of Mitochondrial Membrane Potential in Hair Cells

**Figure 3:**
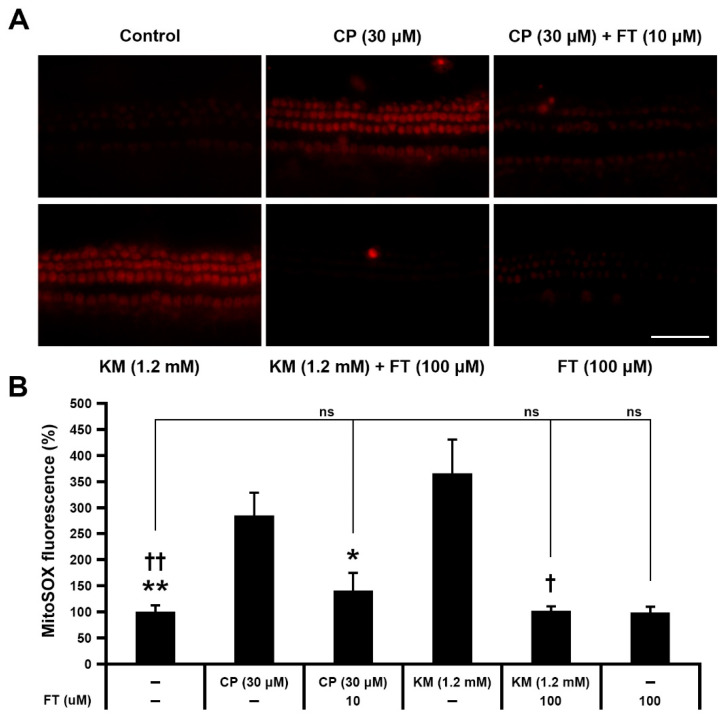
FT prevents mitochondrial ROS accumulation in hair cells of mouse cochlear explants. (**A**) Images of MitoSOX Red immunostaining in Control, CP, CP + FT, KM, KM + FT, and FT groups after 24 h of incubation (*n* = 9, each). Images were acquired from the mid region of the organ of Corti. (**B**) Comparison of red signal intensity ratios between each group. All groups contained 27 mice. Data are shown as mean ± SD. *, ^†^ *p* < 0.05 and **, ^††^ *p* < 0.01 compared with the CP group. CP: cisplatin, KM: kanamycin, FT: fursultiamine, ns: not significant. Scale bar, 50 μm.

Based on previous studies that suggested the CP- and KM-induced ROS generation leading to mitochondrial dysfunction [2,21,22], we analyzed drug-induced accumulation of mitochondrial ROS using MitoSOX Red fluorescence assay. Cochlear explants in the CP and KM groups showed higher fluorescence intensities than those in the CT group, indicating excessive accumulation of mitochondrial ROS (Figure 3A). In contrast, FT pretreatment resulted in decrease of the signal. To quantify fluorescence intensity, we measured the integrated density of red signal, which was expressed as a ratio of 1 for the CT group. The result showed significantly reduced fluorescence intensity in the CP + FT and KM + FT groups compared with the CP and KM group, respectively (*n* = 4 per group, *, ^†^ *p* < 0.05 and **, ^††^ *p* < 0.01; Figure 3B). There were no significant differences between CT and CP + FT or KM + FT groups, showing that mitochondrial ROS level was maintained at the control level despite CP or KM stimulation. It suggests that FT protects hair cells from apoptosis by inhibition of mitochondrial ROS accumulation.

**Figure 4:**
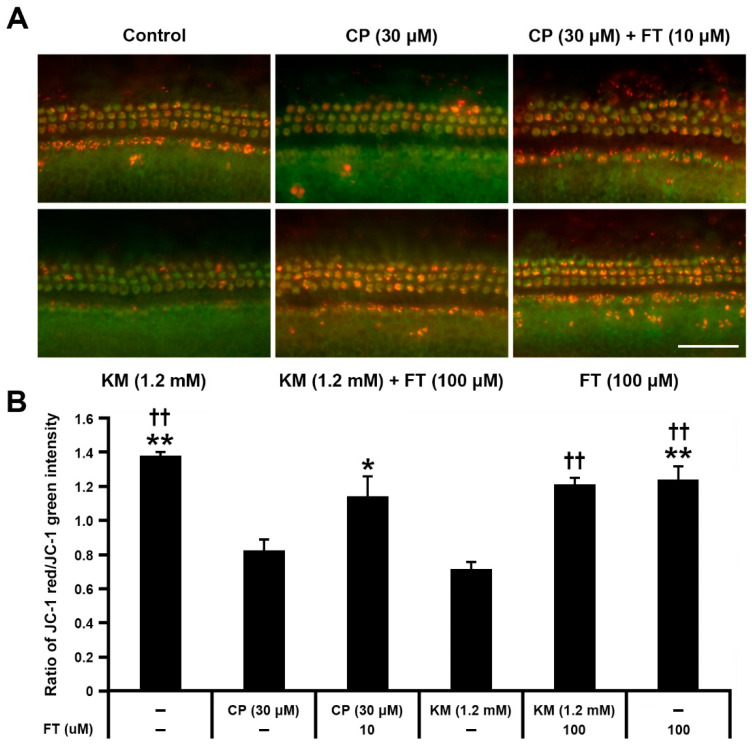
FT inhibits mitochondrial membrane potential disruption in mouse cochlear explants. JC-1 assay exhibiting membrane potential-dependent accumulation of JC-1 aggregates in mitochondria. (**A**) Fluorescence images from Control, CP, CP + FT, KM, KM + FT, and FT groups after 30 h of incubation (*n* = 9, each). Images were acquired from the mid region of the organ of Corti. (**B**) Quantitative comparison of red-to-green fluorescence intensity ratios between each group. All groups contained 27 mice. Data are shown as mean ± SD. * *p* < 0.05 and **, ^††^ *p* < 0.01 compared with the CP group. CP: cisplatin, KM: kanamycin, FT: fursultiamine. Scale bar, 50 μm.

It is well known that mitochondria are the primary source of intracellular ROS, and the organelle susceptible to ROS at the same time [23]. Since depolarization of mitochondrial membrane potential is considered as the initial event of mitochondrial dysfunction [24], we compared the mitochondrial membrane potential between CP or KM-treated cochlear explants with or without FT, using MitoProbeTM JC-1 assay (Figure 4A). Because JC-1 monomers emitting green fluorescence aggregate in mitochondria yielding red fluorescence when the mitochondria maintain high membrane potential, the quantitative analysis of mitochondrial membrane potential was measured in the red/green signal ratio (Figure 4). Compared with the CT group, CP- or KM-treated hair cells showed remarkably decreased red/green ratio with loss of red signal, indicating drug-induced mitochondrial damage. However, when the hair cells were pretreated with FT, the red/green ratio was maintained nearly to the control level in both CP + FT and KM + FT groups. Moreover, this protective effect of FT was statistically significant (*n* = 3 per group, *, ^†^ *p* < 0.05 and **, ^††^ *p* < 0.01; Figure 4B). These results suggest that FT prevents CP and KM-induced dissipation of the mitochondrial membrane potential caused by oxidative stress.

## 4. Discussion

In cochlea, the organ of Corti, stria vascularis, and spiral ganglia are the main areas affected by drug-induced ototoxicity. Although the detrimental effects of CP and KM appears to occur in these regions simultaneously, the hair cells in the organ of Corti have been considered as the primary target of CP and aminoglycoside antibiotics [6,8]. In the present study, we observed severe damage to the organ of Corti in CP- and KM-treated cochlear explants, particularly evidenced by irregular patterns of hair cells. The reduction in hair cell viability was OHC-dominant in the drug-treated cochlear explants, similar to previous studies of drug-induced ototoxicity [25,26]. The more severe ototoxicity in OHCs than in IHCs suggests that IHCs are relatively resistant to these ototoxic drugs. There are multiple differences in extracellular environment and intracellular metabolisms between inner and outer hair cells. Particularly several previous studies demonstrated that cochlear IHCs and OHCs have different expression levels or activities of multiple proteins including transcription factors [27,28]. Particularly in transcriptome analysis of IHCs and OHCs, <15% of all expressed genes were identified to be differentially expressed. It allows us to infer that susceptibility or cellular responses to particular stimuli such as cytotoxic drugs may differ between IHCs and OHCs. For example, Bcl-2 (B-cell lymphoma-2), one of the most important regulators of apoptosis, shows much higher expression level in IHCs than OHCs [27]. This protein exerts anti-apoptotic activity by binding to the pro-apoptotic proteins BAD and BAK, resulting in inhibition of caspase-3-induced apoptosis [29]. Bcl-6, another member of the Bcl-2 family, is also known as an anti-apoptotic protein [30]. This transcriptional repressor also shows higher expression level in IHC, and it has been predicted that Bcl-6 is necessary for survival and functional maintenance of IHCs. Thus, this may provide a clue to why IHCs and OHCs show differential vulnerability to drug-induced hair cell loss.

CP- and aminoglycoside-induced ototoxicity is known to be caused by multiple intrinsic and extrinsic pathways, including direct damage to nuclear or mitochondrial DNA, dysregulation of gene expressions through hypoacetylation of nuclear histones, endoplasmic reticulum (ER) stress, and activation of inflammatory pathway [12]. Among them, excessive production and accumulation of ROS may be the main underlying mechanism, since most of these mechanisms are ultimately linked to ROS. Moreover, both CP and KM increase mitochondrial ROS levels by decreasing the expression and activity of major antioxidant enzymes, such as superoxide dismutase, catalase, glutathione peroxidase, and glutathione reductase [31]. Consequently, the accumulation of ROS exerts direct cytotoxic effects by favoring the opening of the permeability transition pore complex [32]. CP also increases levels of pro-apoptotic transcription factors such as FOXO3a and BAX and decreases Bcl-2 levels, which permeabilize the mitochondrial outer membrane [33,34]. Another study have showed that adenovirus-mediated overexpression of Bcl-XL protected mouse cochlear hair cells from kanamycin ototoxicity [35]. Release of cytochrome C induced by permeabilization of the mitochondrial outer membrane activates intrinsic apoptotic pathways through caspase-9 and caspase-3 [36,37]. Mitochondrial membrane potential is a key indicator of mitochondrial activity, as it reflects the process of electron transport and oxidative phosphorylation, which is the driving force behind ATP production [38]. As the release of mitochondrial factors disrupts mitochondrial membrane potential [39], we specifically examined ROS levels and membrane potential change in mitochondria.

We found that CP and KM increased mitochondrial ROS levels and depolarized mitochondrial membrane potential leading to apoptotic hair cell death, which is highly consistent with other previous studies of drug-induced ototoxicity [40,41]. However, this damage was prevented by pre-treatment with FT, a lipophilic thiamine derivative. Thiamine is required for various cell activities, such as energy production and maintenance of redox balance. The active form of thiamine, thiamine pyrophosphate, is further implicated in negative feedback mechanisms that suppress intracellular p53 activity [42]. Phosphorylated and unphosphorylated thiamine exert protective effects against Wernicke’s encephalopathy, incipient diabetic nephropathy, and free radical-mediated neurotoxicity [43,44,45]. Another previous study proposed that thiamine (i.e., vitamin B1) is a key factor in the auxiliary antioxidant system in the liver of alcoholized rats [19]. Furthermore, FT pretreatment prevents CP-induced reductions in intracellular levels of total glutathione, glutathione S-transferase, glutathione peroxidase, and glutathione reductase in liver tissue [20].

Although thiamine is transported across the plasma membrane of the cell by high-affinity carriers, its transport rate is low. To increase the bioavailability of thiamine, many efforts have sought to develop lipophilic thiamine derivatives, such as benfotiamine and FT. In fact, a pharmacokinetic research shows that administration of FT or benfotiamine increases the concentration of thiamine by 4- or 5-fold in hemolysate [46]. Benfotiamine, a lipid-soluble S-acyl derivative of thiamine, restored CP-induced nephrotoxicity by increasing endogenous antioxidative enzymes in rats [47], and also showed higher bioavailability when it was orally administrated in a clinical study [48]. However, several studies have suggested that benfotiamine is unable to diffuse through the cell membrane unless it is dephosphorylated by ecto-alkaline phosphatases [49,50]. In contrast, when FT contacts the cell membrane, the disulfide is destroyed, the rest of the molecule penetrates the membrane, and the thiazolium ring is closed by a reduction reaction. This non-carrier-mediated transit through cell membranes leads to an accumulation of thiamine in cells [51]. This reaction has the advantage that does not demand the transport system required for intracellular absorption of thiamine [52]. In a previous study, intraperitoneally administrated FT had a strong inhibitory effect on carrageenan-induced paw edema in rats, but this anti-inflammatory effect was not observed for genuine thiamine or its non-disulfide derivatives [53]. Based on these previous studies, we could expect FT to have similar or higher absorptivity and antioxidant effects than benfotiamine, despite controversies over whether benfotiamine is a lipophilic thiamine derivative. Since there has been no reports of ameliorative effects of FT in auditory system, we first investigated preventive effect of FT in drug-induced ototoxicity in this study and identified that FT prevents the CP and KM-induced collapse of mitochondrial membrane potential and increase in mitochondrial ROS in murine cochlear explants. It was consistent with the results of previous studies that were performed using cultured cells or other mammalian organs.

In summary, this study is the first to suggest that the lipophilic thiamine derivative FT functions as a protective agent against anticancer and antibiotic drug-induced ototoxicity and hair cell damage. Our results show that FT may attenuate the accumulation of ROS in mitochondria and ameliorate mitochondrial membrane potential disruption in murine cochlear explants. We speculate that FT acts as an antioxidant and anti-apoptotic agent against CP or KM-induced ototoxicity. To provide a foundation for the clinical application of TF against drug-induced ototoxicity, further in vitro or in vivo studies using CP-sensitive cancer cells or animal models are needed. In fact, mutual influence between antioxidant agents and anticancer activity is highly complicated and controversial [54]. Common antioxidant molecules such as vitamin E and alpha-lipoic acid (ALA) have been known to counteract the anticancer activity of CP by inhibiting ROS-induced apoptosis of cancer cells. It limits their clinical application to prevent CP-induced hearing loss in patients undergoing chemotherapy. In contrast, KL1333 that protects cochlear hair cells from CP ototoxicity by antioxidative effect, is a derivate from β-lapachone that enhances the anticancer activity of CP [55,56]. Another antioxidant, lovastatin that is a statin medication, was reported to reduce CP-induced hearing loss, whereas use of statins including lovastatin are allowed to be used in cancer therapy, because statins could induce apoptosis of cancer cells [57,58]. Thus, it is necessary to further investigate how FT works in cancer cells to estimate if FT has value for clinical use to prevent drug-induced hearing loss.

## Data Availability

The data are contained within the article.

## Supplementary Materials

The following are available online at https://www.mdpi.com/article/10.3390/antiox10101526/s1, Figure S1. High-magnification images of hair cell stereocilia in mouse cochlear explants from Control, CP, CP + FT, KM, KM + FT, and FT groups.
