# Supplementary material for: Fursultiamine Prevents Drug-Induced Ototoxicity by Reducing Accumulation of Reactive Oxygen Species in Mouse Cochlea
Source: Antioxidants (Basel). 2021 Sep 26;10(10):1526. doi: 10.3390/antiox10101526 (PMC8533091; doi:10.3390/antiox10101526)
Supplement: Supplementary file 1 [file antioxidants-10-01526-s001.zip › antioxidants-1354174-supplementary.pdf]

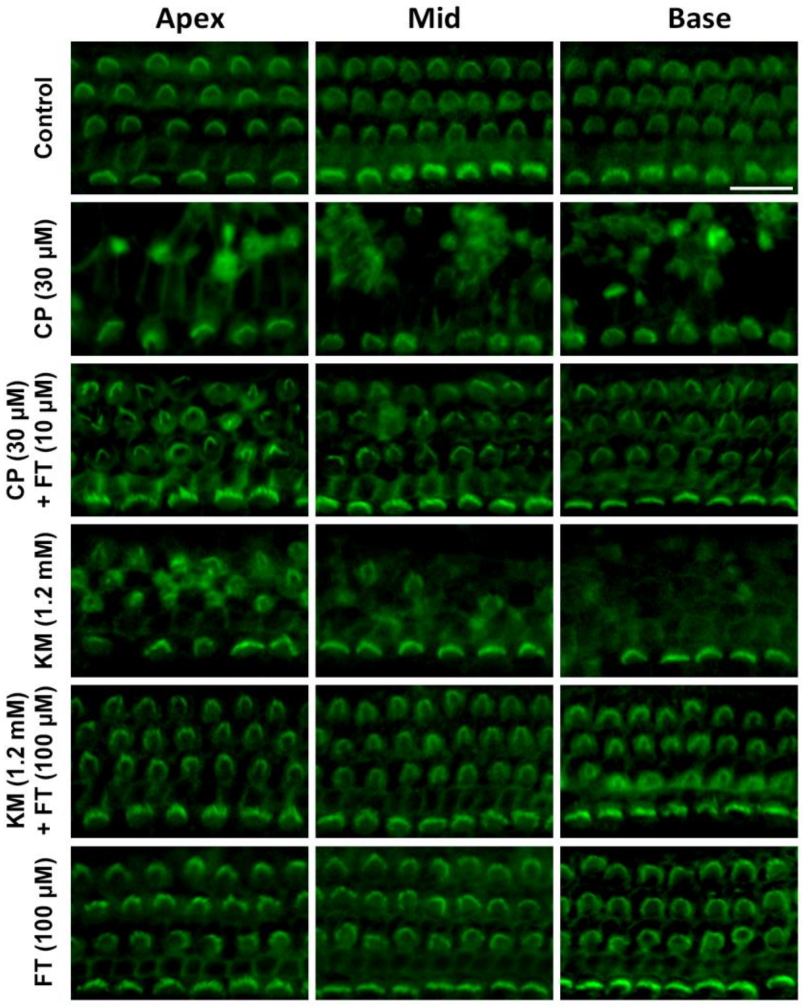

Figure S1. High-magnification images of hair cell stereocilia in mouse cochlear explants from Control, CP, CP + FT, KM, KM + FT, and FT groups.
